# Towards Controlling the Local Bone Tissue Remodeling—Multifunctional Injectable Composites for Osteoporosis Treatment

**DOI:** 10.3390/ijms24054959

**Published:** 2023-03-04

**Authors:** Joanna Klara, Sylwia Onak, Andrzej Kowalczyk, Wojciech Horak, Kinga Wójcik, Joanna Lewandowska-Łańcucka

**Affiliations:** 1 Faculty of Chemistry, Jagiellonian University, Gronostajowa 2, 30-387 Kraków, Poland; 2 Department of Machine Design and Technology, Faculty of Mechanical Engineering and Robotics, AGH University of Science and Technology, Al. Mickiewicza 30, 30-059 Kraków, Poland; 3 Department of Microbiology, Faculty of Biochemistry, Biophysics and Biotechnology, Jagiellonian University, Gronostajowa 7, 30-387 Kraków, Poland

**Keywords:** multifunctional composites, biopolymers, mesoporous silica particles, hydrogels, alendronate, drug delivery systems, osteoporosis

## Abstract

Alendronate (ALN) is the most commonly prescribed oral nitrogen-containing bisphosphonate for osteoporosis therapy. However, its administration is associated with serious side effects. Therefore, the drug delivery systems (DDS) enabling local administration and localized action of that drug are still of great importance. Herein, a novel multifunctional DDS system based on the hydroxyapatite-decorated mesoporous silica particles (MSP-NH_2_-HAp-ALN) embedded into collagen/chitosan/chondroitin sulfate hydrogel for simultaneous osteoporosis treatment and bone regeneration is proposed. In such a system, the hydrogel serves as a carrier for the controlled delivery of ALN at the site of implantation, thus limiting potential adverse effects. The involvement of MSP-NH_2_-HAp-ALN in the crosslinking process was established, as well as the ability of hybrids to be used as injectable systems. We have shown that the attachment of MSP-NH_2_-HAp-ALN to the polymeric matrix provides a prolonged ALN release (up to 20 days) and minimizes the initial burst effect. It was revealed that obtained composites are effective osteoconductive materials capable of supporting the osteoblast-like cell (MG-63) functions and inhibiting osteoclast-like cell (J7741.A) proliferation in vitro. The purposely selected biomimetic composition of these materials (biopolymer hydrogel enriched with the mineral phase) allows their biointegration (in vitro study in the simulated body fluid) and delivers the desired physicochemical features (mechanical, wettability, swellability). Furthermore, the antibacterial activity of the composites in in vitro experiments was also demonstrated.

## 1. Introduction

Osteoporosis is considered as one of the most progressive, systemic, and metabolic diseases affecting bone tissue [1]. It can occur not only due to the genetic predisposition, but also as the effect of metabolic, immunological and hormonal disbalances. Osteoporosis is manifested by reduced bone mass and microarchitectural deterioration as a result of the decoupling of the resorption and bone formation processes [2]. Disturbed skeletal homeostasis can enhance predisposition to osteoporotic-based bone fractures, which can occur even after small traumas, and further decrease the quality of life of patients. According to the World Health Organization (WHO), accelerated bone loss, especially in older people, makes osteoporosis a serious public health problem [3]. Statistics indicate that one in three women and at least one in six men will suffer from osteoporosis-like fractures in their lifetime. Moreover, reports show that over 23 million people in the EU are at high risk of developing osteoporosis, making this disease a major socio-economic problem. Treatment/rehabilitation of millions of osteoporotic breakages costs European healthcare systems more than EUR€ 56 billion per year (data for 2019) [4].

Alendronate (ALN), a nitrogen-containing bisphosphonate, is the leading drug in the treatment of osteoporosis. ALN acts by inhibiting farnesyl pyrophosphate (FPP) synthase, the main enzyme of the mevalonate pathway, thereby interfering with osteoclast activity, including migration, attachment, and resorption, leading to cell death by apoptosis. Unfortunately, being a BCS III drug (high solubility and low permeability due to a polar hydrophilic nature), ALN is characterized by limited oral absorption and bioavailability (0.9–1.8%) [5]. Thus, the high oral doses that need to be taken may cause a range of adverse effects, including upper gastrointestinal tract irritation (indigestion, esophagitis, vomiting, nausea, abdominal pain), as well as severe musculoskeletal pain and cardiovascular risk [6]. Additionally, the specific method of drug administration could be seen as another disadvantage associated with oral delivery. In order to avoid a further decrease in absorption and bioavailability, patients are advised to take ALN 30–60 min before the first meal, and if they want to ingest a meal with divalent metal ions, the time is even longer—up to 2 h [7]. Additionally, to limit irritation of the esophagus, a standing position should be maintained after 30 min of drug administration. Effectiveness in hip, forearm, and spine fracture prevention was described; however, osteonecrosis of the jaws (BRONJ) could be developed as an undesirable side effect [8,9]. ALN administered intravenously also caused serious side effects including fever, flu symptoms, electrolyte imbalance, as well as the risk of nephrotoxicity [10]. Moreover, the changes in bone mineral density (BMD) and vertebral fracture prevention were not superior [11].

Therefore, the approach based on the delivery of ALN specifically to the diseased bone tissue seems to be an extremely promising solution. Combining alendronate with nano/micro materials and developing bone-specific drug delivery systems (DDS) can not only eliminate side effects but also improve the overall effect of therapy. Various types of DDS for alendronate delivery have been presented in the literature [12]. Among them, nano/microformulations [13], polymer conjugates/composites, calcium phosphate ceramics-based materials [14], hydrogel [3], scaffolds [15], coated-like structures, as well as organic-inorganic hybrids have been developed. It was reported that ALN, while encapsulated, is easily lost in the aqueous phase and uncontrollably diffused out from the carrier. An interesting alternative are the injectable hydrogel-based DDS, enabling non-invasive placement at the implantation site. However intrinsic permeability and limited interactions between low-molecular weight drugs and the polymeric matrix make it difficult to ensure sustained delivery and avoid a burst release. To minimize these limitations, the hydrogels are reinforced with different nano/microparticles and hybrid systems for controlled ALN delivery are fabricated [16,17]. In our previous work, a hybrid-based system with silica particles decorated with HAp and with alendronate attached utilizing ALN-HAp affinity was presented [18]. The physicochemical investigations demonstrated the conjugation of alendronate to bioactive silica-hydroxyapatite particles; however, the weight content of the anchored ALN was only about 3.3%.

Herein mesoporous silica particles (MSP) are proposed as a more effective delivery system with excellent features such as a high loading capacity, surface modification possibility, as well as biocompatibility [19]. On the other hand, the high porosity lowers the mechanical properties of a carrier and may contribute to an initial burst or premature release, leading to supraphysiological levels of a drug. In addition, pristine MSP will not ensure the local delivery of the drug. To overcome these limitations, the novel methodology based on the carriers in the form of mesoporous silica particles functionalized with amino groups, decorated with hydroxyapatite and loaded with alendronate (MSP-NH_2_-HAp-ALN), in conjunction with hydrogel-based biomaterials, is proposed within this work. To the best of our knowledge, this approach has not been presented so far. One can assume that such designed materials will serve as an injectable system for the non-invasive delivery of ALN, ensuring satisfactory encapsulation efficiency, localization at the implantation site, and thus limiting side effects. Furthermore, we hypothesize that the incorporation of ALN in the form of MSP-NH_2_-HAp-ALN dispersed in chemically crosslinked hydrogels will minimize an initial burst release of the drug.

MSP decorated with hydroxyapatite will, at the same time, possess bone regeneration activity resulting from osteogenic HAp features. Hydroxyapatite (HAp) closely resembles the mineralized phase of bone and tooth. HAp is a bioactive material and is able to bind to the recipient’s tissue after in vivo implantation. Additionally, it has a high affinity for alendronate [18]. In order to mimic the composition of a natural extracellular matrix (ECM) and thus support the cell’s functionality, native-like and abundant biopolymers have been selected for hydrogels fabrication, namely: collagen (Col), chondroitin sulfate (CS), and chitosan (Ch). Collagen is the main component of ECM, as well as bones. Chondroitin sulfate (CS) is the representative of GAGs (glycosaminoglycans) and can stimulate the synthesis of collagen II, as well as bind calcium and calcium phosphates, thus supporting the local osteoblast adhesion. Chitosan is a linear polysaccharide that accelerates wound healing and possesses antibacterial and antifungal properties [20].

The interaction of biomaterials with not only bone, but also with other body systems is an important and very complex topic. When a scaffold is placed in the body, it can trigger a negative immune response, which can lead to implant failure and even tissue damage. This can limit the effectiveness of applied bone tissue engineering materials and result in overall poor clinical outcomes. Additionally, the immune system plays a crucial role in bone regeneration by facilitating the removal of damaged tissue, as well as promoting the growth of the new one. Mesenchymal stem cells (MSCs) have been shown to have immunomodulatory effects and, by interacting with immune cells and regulating the immune response, MSCs can help to prevent material rejection and promote tissue repair by creating a more favourable microenvironment for bone formation and regeneration. Moreover, biophysical and biochemical stimuli can influence immune cells and promote MSCs osteogenesis [21,22]. Considering bone implantation, it should also be emphasized that infection is a serious problem for healthcare systems with significant socio-economic implications. When bone tissue is infected, the administration of antibiotics is necessary. The main challenge then is to deliver the pharmaceuticals to the infected tissue, which is difficult if employing conventional therapies [2,23]. The composites developed within this work also addressed this issue. It is expected that systems of the proposed herein composition (the polymeric matrix with chitosan added) will possess the antibacterial features that will enable it to minimize and to neutralize the bacteria action without the need for antibiotics treatment.

The main disadvantage associated with hydrogels is their poor mechanical properties. One solution to overcome this problem is the incorporation of inorganic particles [24]. Moreover, to obtain hybrids with satisfactory mechanical stability and degradation profiles, the chemical crosslinking might be also applied. To fabricate the structurally stable composites, we have utilized the genipin, a non-toxic crosslinking agent of natural origin that reacts with amino groups while crosslinking. Importantly the approach presented herein is based on the use of MSP particles modified with NH_2_- groups, which makes possible the covalent attachment of the resulted MSP-NH_2_ to the polymeric hydrogel network during crosslinking with genipin. That procedure is of great importance as it allows for the preparation of the composites in which the potential phase separation of mineral particles, hindering in vivo application, is eliminated. Moreover, to ensure the effective crosslinking of all used biopolymers the chondroitin sulfate was also functionalized with amino groups according to the protocol developed by us previously [20].

Hydrogel-based composites with three different concentrations of MSP-NH_2_-HAp-ALN were fabricated and characterized for physicochemical and biological properties. The swelling, wettability, microstructure, mechanical characteristics, as well as enzymatic degradation were evaluated. To demonstrate the impact of mesoporous silica-based particles on the bioactivity of the resulted materials, the in vitro biomineralization employing a simulated body fluid model (SBF) was performed. Preliminary in vitro biological studies were carried out using model osteoblast-like and osteoclast-like cells. Furthermore, to demonstrate the multifunctionality of developed composites, their antibacterial activity against *Escherichia coli* and *Staphylococcus aureus* strains was also evaluated.

## 2. Results and Discussion

### 2.1. The Functionalized Mesoporous Silica-Based Particles Decorated with Hydroxyapatite and Loaded with Alendronate (MSP-NH_2_-HAp-ALN)—Synthesis and Characterization

**Figure 1:**
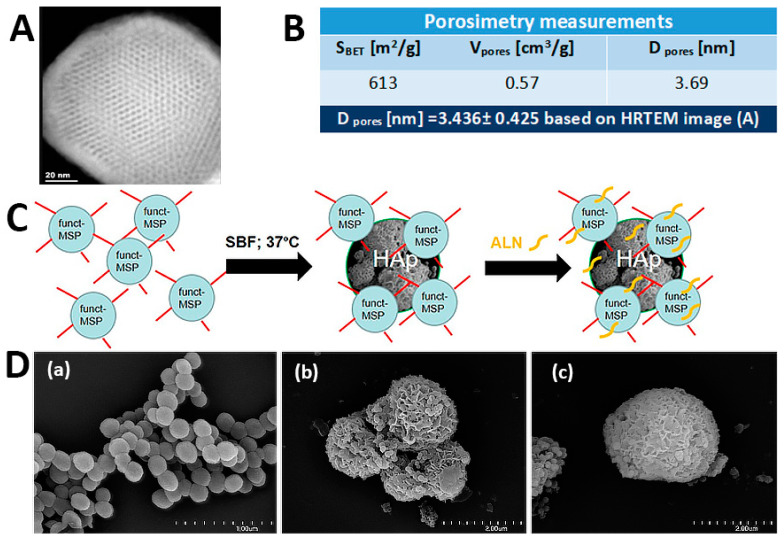
(**A**) HRTEM image for MSP-NH_2_ particles, the scale bar represents 20 nm. (**B**) Data from porosimetry measurements, and HRTEM pore analysis. (**C**) Scheme of the synthesis of the functionalized mesoporous silica-based particles decorated with hydroxyapatite (HAp) and loaded with alendronate (ALN) (MSP-NH_2_-HAp-ALN). (**D**) SEM microphotographs for (**a**) MSP-NH_2_, (**b**) MSP-NH_2_-HAp, and (**c**) MSP-NH_2_-HAp-ALN particles; the scale bars represent 1 µm for (**a**) and 2 µm for (**b**,**c**).

**Figure 2:**
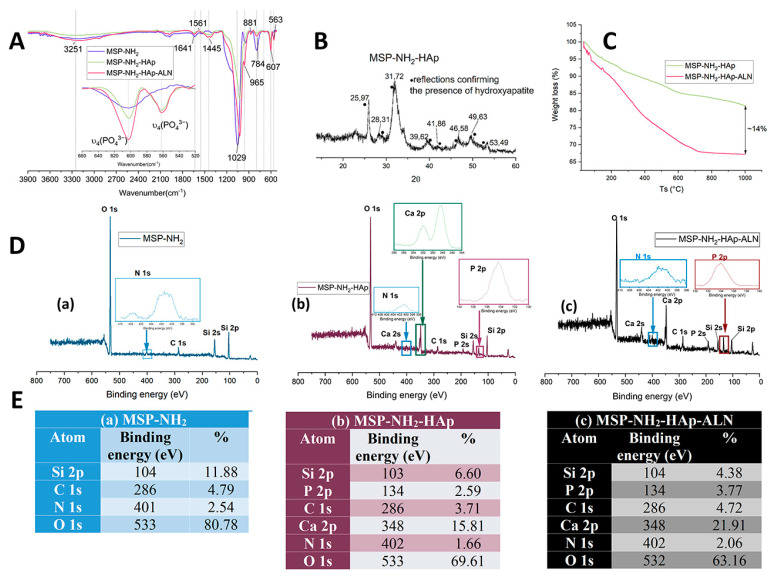
(**A**) FT-IR spectra for MSP-NH_2_, MSP-NH_2_-HAp, and MSP-NH_2_-HAp-ALN particles. (**B**) XRD for MSP-NH_2_-HAp. (**C**) Thermogravimetry curves for MSP-NH_2_-HAp and MSP-NH_2_-HAp-ALN particles. (**D**) XPS spectra for (**a**) MSP-NH_2_, (**b**) MSP-NH_2_-HAp, and (**c**) MSP-NH_2_-HAp-ALN particles. (**E**) Calculated peak area and atom percentage in (**a**) MSP-NH_2_, (**b**) MSP-NH_2_-HAp, and (**c**) MSP-NH_2_-HAp-ALN particles.

The synthesis of mesoporous silica nanoparticles is generally based on the modified Stöber method, which initially yielded micron-sized monodispersed and non-porous silica spheres. The addition of surfactants results in the fabrication of silica nanoparticles with excellent physicochemical features and high porosity. Importantly, by controlling the reaction conditions (e.g., pH, temperature, surfactant concentration), the preparation of homogenous nanoparticles within the range 50–300 nm is possible. The surface of MSP presents a high density of silanol groups, which brings the option of further modification utilizing organosilanes bearing various functional moieties (amino, carboxylic acid, thiol etc.) [25]. Herein, the direct functionalization based on the addition of a trialkoxysilane with the selected group (-NH_2_) to the reaction mixture during the MSNs synthesis was employed. Utilization of CTAB surfactant during synthesis as a template allowed for the creation of well-ordered pores with a diameter of 3.436 ± 0.425 nm based on the HRTEM (High-resolution transmission electron microscopy) image (Figure 1A), which resulted in high porosity. Additionally, based on the nitrogen adsorption study, the pore size diameter and pore volume were established as 3.7 nm and 0.57 cm^3^/g, respectively (Figure 1B). This pore diameter, according to the literature [26], is in the size range characteristic for mesoporous structures. The total BET surface in obtained particles was equal to 613 m^2^/g. The successful functionalization of MSP with amino groups was proven by XPS measurements (Figure 2(Da)) and elemental analysis. The presence of the peaks around 401 eV (N 1s) and 286 eV (C 1s) are related to the used APTES ((3-Aminopropyl)triethoxysilane) precursor, which was the donor of the NH_2_ groups. Additionally, the amino group percentage present in the surface layer of MSP-NH_2_ particles was equal to 2.5%, based on the nitrogen content, which is close to the value obtained from elemental analysis—2.2%.

The formation of amino-functionalized mesoporous silica–based particles decorated with hydroxyapatite (HAp) and loaded with alendronate (ALN) is schematically illustrated in Figure 1C. SEM (Scanning electron microscopy) microphotographs of resulting MSP-NH_2_, MSP-NH_2_-Hap, and MSP-NH_2_-HAp-ALN particles are presented in Figure 1(Da), Figure 1(Db), and Figure 1(Dc), respectively. The incubation in 1.5× SBF (simulated body fluid) at 37 °C was performed to decorate the obtained mesoporous silica particles functionalized with amino groups with the mineral phase. EDS (Energy-dispersive X-ray spectroscopy) analysis revealed that after 5 days of the experiment, the mineral plate-like phase with a Ca/P ratio equal to 1.1 was present on particles (Figure 1(Db)). Additionally, the presence of these elements was also revealed on the XPS (X-ray photoelectron spectroscopy) spectrum (Figure 2(Db)) around 348, 352 eV (15.81%), and 134 eV (2.59%) for Ca 2p and P 2p, respectively. Negatively charged groups, especially OH groups in MSP, create an environment supporting PO_4_^3−^ and Ca^2+^ ion nucleation on the surface of the particles [27,28,29,30].

FT-IR (FourierTransform Infrared Spectroscopy) spectra depicted in Figure 2A confirmed the presence of PO_4_^3−^ ions (607, 553 cm^−1^). The bands at 3251 cm^−1^ (OH stretching), 1641 cm^−1^ and 1561 cm^−1^ (N-H bending), 1445 cm^−1^ (CH_2_ bending), 1029 cm^−1^ (asymmetric Si-O-Si stretching), 965 cm^−1^ (Si-OH), 881 cm^−1^ (asymmetric Si-O-Si stretching), and 784 cm^−1^ (symmetric Si-O-Si stretching), originate from pure mesoporous silica functionalized with amino groups [31]. Additionally, in order to establish whether the obtained mineral phase is hydroxyapatite, XRD analysis was performed (Figure 2B). The obtained diffractogram was compared with the literature [32,33], and reflections at 2θ of approximately 26, 28.3, 31.7, 39.7, 46.6, 49.6, and 53.5 confirmed the successful HAp formation under the employed synthesis conditions. Loading of the alendronate into MSP-NH_2_-HAp particles was accomplished after 3 days of reaction in an alkaline solution at 37 °C.

Based on the recorded XPS spectra and the calculated elemental percentage (Figure 2D,E), the incorporation of alendronate into mesoporous silica-based particles can be postulated. The noticeable increase in the percentage of N 1s (401 eV) and P 2p (134 eV) atoms between MSP-NH_2_-HAp and MSP-NH_2_-HAp-ALN structures may be related to the presence of ALN. As the alendronate has similar groups to hydroxyapatite (PO_4_^3−^) and to MSP-NH_2_ (NH_2_), the FT-IR spectrum of the resulting drug-loaded particles (Figure 2A) has not revealed any significant differences. In order to verify the degree of drug loading, a thermogravimetry study was conducted. Figure 2C shows the weight loss for MSP-NH_2_-HAp and MSP-NH_2_-HAp-ALN samples. In our previous work, we have presented the thermogram of silica particles with two characteristic mass loss steps connected with the removal of the physically adsorbed water (up to 130 °C) and the slow condensation of silanols (above 190 °C), respectively. The estimated total mass loss was equal to 11%. Herein amine-functionalized mesoporous silica particles decorated with HAp were utilized, so the weight change was higher (about 18% for MSP-NH_2_-HAp), since it also originated from the propyl groups introduced via APTES. The TG (thermogravimetry) profile for HAp revealed only 1% weight loss [18]. Overall, comparing TG profiles obtained for MSP-NH_2_-HAp and MSP-NH_2_-HAp-ALN materials, and based on the final mass percentage, the amount of alendronate in prepared structures was established as 14%.

### 2.2. Hybrid Systems—Physicochemical Characterization

In our previous work [20], we developed novel collagen/chitosan/lysine-functionalized chondroitin sulfate (ColChCS_mod_) injectable hydrogels for tissue engineering applications. Our findings demonstrated CS_mod_-content-dependent bioactivity of obtained systems without the need for applying additional inducers. Herein, the hydrogel composed of Col, Ch, and CS_mod_ (wt% 50:20:30) crosslinked with 20 mM genipin solution was selected as a bioactive and biocompatible matrix for MSP-NH_2_-HAp-ALN delivery with the superior swelling, degradability, and mechanical features. We studied three types of hybrid systems, with different amounts of particle concentration—HybC1 (0.5 mg/mL), HybC2 (1.25 mg/mL), and HybC3 (2.5 mg/mL). Hydrogel without particles was used as the reference material.

**Figure 3:**
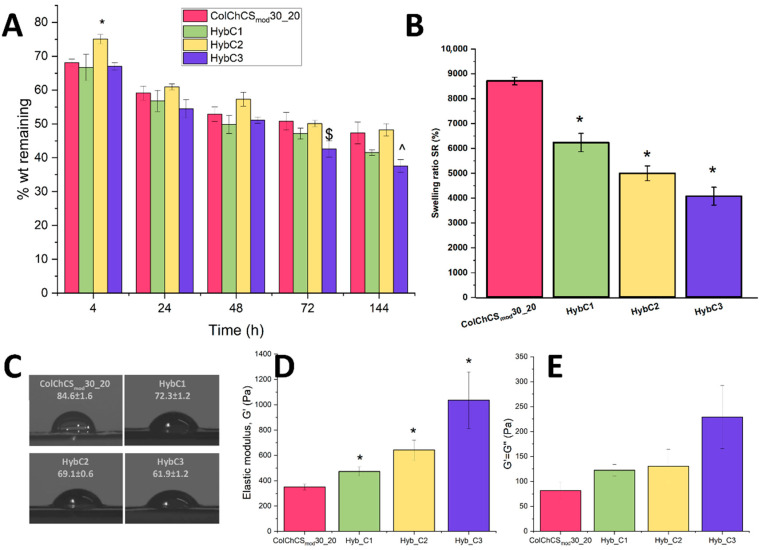
(**A**) Enzymatic degradation for prepared hybrid systems and hydrogel ColChCS_mod_30_20. *, $, ^ indicates statistical significance when compared with ColChCS_mod_30_20 after 4 h, 72 h, and 144 h of experiment. (**B**) Swelling ratio for prepared hybrid systems and ColChCS_mod_30_20. * indicates statistical significance when compared with ColChCS_mod_30_20. (**C**) Images and contact angle values (in degrees) for prepared hybrids and hydrogel samples. (**D**) Values of the elasticity modulus. * indicates statistical significance when compared with ColChCS_mod_30_20. (**E**) The result of the measurement of parameters characterizing the flow point (G′ = G″) for the created hybrid systems and ColChCS_mod_.

The ability of materials to maintain their mass and to resist complete degradation is an important factor to consider when systems are intended for bone tissue engineering. However, to eliminate the possibility of the stress-shielding effect and to enable the transfer of biological stress to the created new bone tissue, prepared systems should exhibit some degree of degradation tendency [34]. It is expected that the process of materials’ biodegradation will be adapted to the rate of new tissue formation [35]. The presented herein degradability study (Figure 3A) was therefore conducted with the utilization of collagenase enzyme, as the main component of prepared hybrid systems was collagen. The high concentration of this compound in comparison to the concentration present in the inflamed organism [36], allowed for an accelerated experiment in which the degradability of prepared hybrids and hydrogel was assessed.

The observed tendency for all tested samples is similar, and materials are gradually degraded. The incorporation of MSP-NH_2_-HAp-ALN did not significantly decrease the resistance of hybrid systems to undergo biodegradation. All of the prepared materials, after 6 days of the experiment, maintained more than 35% of their initial mass. The lowest mass loss after this time was observed for HybC2 and ColChCS_mod_30_20 (~48% of weight remaining). Hybrids with the highest particle concentration were more susceptible to the degradation with collagenase enzyme (37% of initial weight after 6 days, statistical significance when compared with ColChCS_mod_30_20). However, there is no clear correlation between the amount of used inorganic component and the tendency to degrade, as the system with the lowest MSP-NH_2_-HAp-ALN concentration (HybC1) maintained only 41% of its initial mass. Overall, based on the obtained results and taking into account the experimental conditions, it seems that the composition of the polymeric matrix is the main factor affecting the degradation process.

When considering materials for bone tissue engineering, several aspects must be considered to allow for efficient bone formation. One of these factors could be evaluated by performing swellability tests. The ability of materials to swell is related to their mesh size, which in turn might influence both nutrient transport and drug delivery, and therefore affect cell proliferation and differentiation [37,38,39]. As can be seen in Figure 3B, pristine hydrogel exhibits a significant swelling ratio (SR = 8711), as the used polymers have a great abundance of hydrophilic groups (OH, -NH_2_, -COOH, and -SO_3_H) that are capable of water-binding without any dissolving of its polymeric components [40,41]. The addition of MSP-NH_2_-HAp-ALN particles into the hydrogel matrix significantly decreased the value of SR, and the effect was correlated with the concentration of used particles. The lowest swelling ratio (SR = 4062) was found for the hybrid system with the highest quantity of particles (HybC3), which is almost two times lower than for hydrogel samples. This observation is in agreement with other studies utilizing silica particles [18,42,43,44], in which a similar tendency was observed. The MSP-NH_2_-HAp-ALN particles occupy the free spaces within the crosslinked matrix, and thus the water uptake in the resulting hybrid systems is hampered when compared to the pure hydrogel. Overall, the particle content-dependent decrease in the swelling ability of developed hybrids was established.

For all prepared hybrid systems, as well as for ColChCS_mod_30_20, the wettability studies were performed by the means of contact angle measurements (Figure 3C). The hydrophilicity of materials prepared for bone tissue engineering influences their applicability and ability to successfully adhere cells, as well as their proliferation [45,46]. For example, the increased wettability in the Ti surface allowed for improved osseointegration in the presence of osteoporosis [47], and enhanced bone formation by regulating angiogenesis, bone mineralization, and bone remodeling [48,49]. The hydrogel sample is characterized by moderate wettability, as its contact angle is equal to 84.6. A decrease in this value was noticed even for the hybrid with the lowest particle content (72.3 ± 1.2 for HybC1). When the particle concentration was increasing, the contact angle was decreasing, making hybrids more hydrophilic—the lowest value was measured for HybC3 equal to 61.9 ± 1.2. This observation could be explained by the high abundance of hydroxy (OH) groups present in particles, both derived from hydroxyapatite, alendronate, and the particles of mesoporous silica functionalized with amino groups, as well as other hydrophilic groups. The revealed herein trend is in agreement with other studies—the addition of hydroxyapatite in the PLGA matrix [50], silica particles into the hydrogel matrix [18], and alendronate into the gelatin methacryloyl hydrogel [51] also improved their hydrophilicity.

### 2.3. Injectability and Rheological Evaluation

Composites developed within this work are considered to serve as injectable systems that will be introduced to the osteoporotic-like bone defects by viscous sol injection, and subsequently in situ gelated under physiological conditions. That way of administration is of great interest since the minimal scarring, the ease of operation, and thus increased patient comfort are delivered. To verify that concept, the injectability experiment in situ was performed. Hybrid in sol state was drawn into the syringe and was then injected through a 27G needle into the Eppendorf tube. The sample was subsequently incubated at 37 °C, and after 5 min, the gel was formed (non-flowable) and the composite was no longer capable of undergoing injection. Additionally, after 24 h of incubation at 37 °C, the gels change color from milky white to dark greyish-blue cream, which is characteristic for the genipin-involved crosslinking. We present this experiment in the form of a short video that could be found in the Supplementary Materials. As was demonstrated, designed systems are injectable since the gel is formed even after injection. The ability to pass the 27 G needle is related to the free-fluid features of the prepared sol. The crosslinking process is initiated at 37 °C with genipin (crosslinking agent) action via NH_2_ groups in polymers and MSP-NH_2_-HAp-ALN particles, thus the gelation process occurs and the sample starts to have solid-like properties.

The response of materials, when the stress was applied, was studied using the rheological measurement in the oscillatory mode. All tested samples in the form of gel have been subjected to Amplitude Sweep (AS) tests, for strain in the range γ = 0.1–1000% and the frequency f = 1. By utilization of experiments with constant frequency and variable strain amplitude, the area of LVE (linear viscoelastic range) in which the magnitude of imposed strain does not influence the properties of materials was established as γ ≤ 5%. The recorded values of elastic modulus (G′) read at 1% strain and the flow point in which G′ = G″ are presented in Figure 3D and Figure 3E, respectively. G′ is the measure of the sample’s stiffness, whereas G″ is representative of the flow or liquid-like response of the material. When G′ > G″, the sample behaves similarly to an elastic solid, while for G″ > G′, the sample exhibits viscous liquid-like features [52]. For all tested materials, G′ values were significantly higher than G″ values, which proves that the obtained hybrid materials, as well as the pristine hydrogel, show the advantage of elastic properties over viscous properties resulting from the effective crosslinking. Moreover the substantial impact of the MSP-NH_2_-HAp-ALN content on the G′ values was observed. It was revealed that the incorporation of particles increased the elastic modulus of the resulted composites (statistical significance when comparing with G′ values for ColChCS_mod_30_20) (Figure 3D), and the effect was higher for the hybrids with higher MSP-NH_2_-HAp-ALN concentration. This parameter is strictly related to the stiffness of materials, and that in turn is linked with the crosslinking degree. As the MSP-NH_2_-Hap-ALN concentration is higher, the resulting gel is more stiff, and its mechanical properties are improved almost three-fold (G′ = 1035 Pa for HybC3) compared to the ColChCS_mod_ hydrogel; G′ is equal to 350 Pa. Additionally, the so-called flow point in which the curve for the elastic and loss modulus are crossed (G′ = G″) occurred in higher values for hybrid systems (see Figure 3E). A clear tendency can be indicated that, with the increase in the particle content, the value of the crossover point occurs at higher values. This means that the hybrid materials are characterized by a higher average flow stress compared to the pristine hydrogel, thus showing greater resistance to mechanical damage. Overall, it was found that the incorporation of inorganic particles into the hydrogel matrix improved its mechanical properties and the MSP-NH_2_-HAp-ALN content-dependent effect was revealed.

### 2.4. Biomineralization

**Figure 4:**
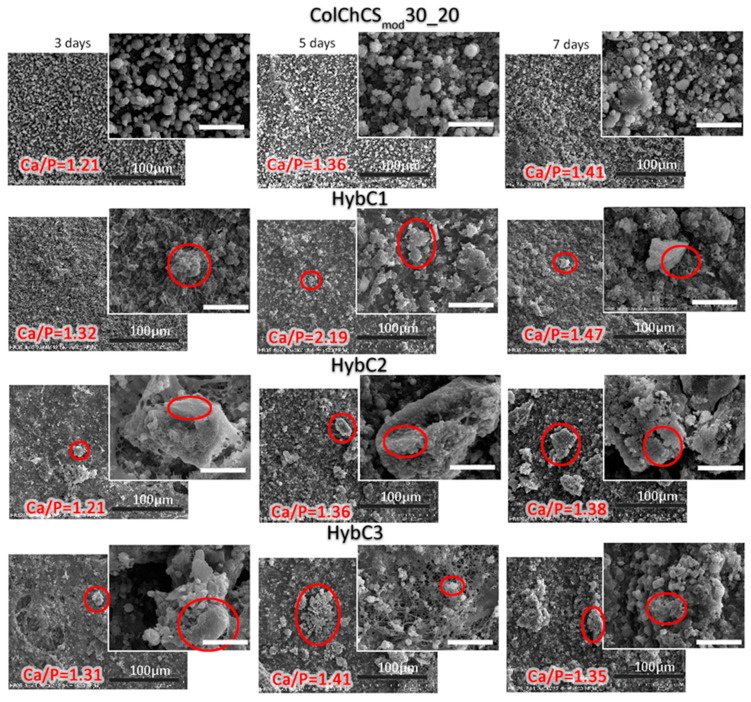
SEM microphotographs obtained after biomineralization experiments lasting 3, 5, and 7 days. The scale bars (black) in the main images represent 100 µm, while they are 20 µm (white) in the inset images. For Ca/P calculation, the EDS analyses in different areas of the samples were conducted, and the presented Ca/P ratio is an average value. The new mineral phases formed on the hybrids are marked with the red circles.

Taking into account the potential application of the developed composites for TE, we have carried out a model of in vitro biomineralization in simulated body fluid (SBF) conditions. It was established that the biointegration of material in vivo can be followed by sample incubation in SBF [53]. Therefore, the ability of developed materials to stimulate the formation of a new mineral phase in the SBF environment was assessed for 3, 5, and 7 days of incubation at 37 °C. Next, the tested samples were analyzed utilizing SEM and EDS techniques, and the resulting microphotographs and calculated Ca/P ratio are depicted in Figure 4. For all materials, both composites and pristine hydrogel, the formation of new mineral phases after 3 days of experiment was revealed. EDS analysis of the created tightly packed crystallites proved the presence of calcium and phosphorus atoms with the ratio characteristic for an apatite-like structure. In our previous work [20], we assessed the capability of prepared hydrogels to act as bioactive materials. We have found that the addition of modified chondroitin sulfate in collagen-chitosan hydrogels improved the bioactivity of tested systems, and the effect was correlated with the amount of CS_mod_ used. We have demonstrated that the ColChCS_mod_30_20 matrix itself has the ability to induce mineralization. Herein, it can be also seen that, for pure hydrogel, both the range of the mineral phase and the value of the Ca/P ratio increased as the experiment was prolonged. The Ca/P ratio changes from 1.21 to 1.41 between the 3rd and 7th day. The mineral phases appeared on the surface of the hydrogel in the form of cauliflower-like individual objects. For developed composites, a different mineralization process can be observed since new phases appeared after 3 days of incubation and were in the form of irregularly shaped individual aggregates (see the structures marked in red in Figure 4). We found that the extended incubation time is not directly related to the obtained Ca/P ratio. The Ca/P ratios after 3 and 7 days of incubation are in the ranges 1.32–1.47, 1.21–1.38, and 1.31–1.35 for HybC1, HybC2, and HybC3, respectively. They are close to the ratios in compounds such as octacalcium phosphate (OCP, Ca/P ratio of 1.33) and tricalcium phosphate (TCP, Ca/P ratio of 1.5) [18]. Interestingly, for the composite with the lowest concentration of particles, a large change in the Ca/P ratio between 3 and 5 days was revealed (from 1.32 to 2.19). This high Ca/P ratio may result from the coexistence of CaO formation together with another mineral phase. Moreover, it was observed that a higher concentration of particles in the systems favored the formation of larger aggregates. The creation of more complex structures is especially pronounced for HybC2 and HybC3 composites. For effective apatite formation, materials should possess the active groups that play a key role in its nucleation. We have found that functionalized chondroitin sulfate with anionic groups (sulfate and carboxyl) serves as the effective calcium ion binding sites, thereby supporting the calcium phosphate nucleation on the surface of ColChCS_mod_ hydrogels [20]. Furthermore, the composites developed in this study were prepared with a widely known bioactive element in the form of hydroxyapatite in MSP-NH_2_-HAp-ALN particles, which is also the main component of the mineral phase of the bone [35,54]. To sum up this aspect of our research, one can conclude that the presence of MSP-NH_2_-HAp-ALN particles in the ColChCS_mod_ matrix supports the mineralization process, which will ensure more effective biointegration in vivo.

### 2.5. Model In Vitro Release Study

**Figure 5:**
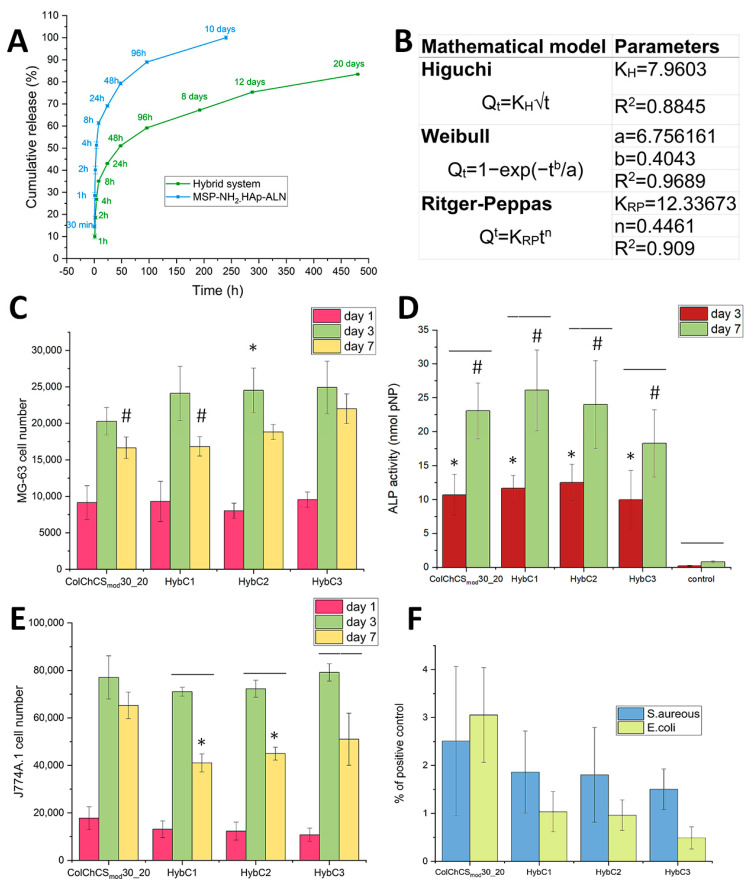
(**A**) Release curve for the hybrid system and MSP-NH_2_-HAp-ALN particles, expressed as the percentage of total ALN released from MSP-NH_2_-HAp-ALN particles. (**B**) Mathematical kinetic model parameters for release from the hybrid system; R^2^—the coefficient of determination Q_t_—the amount of drug released in time t, K_H_, K_RP_—the release rate constants, n—the release exponent, a—the scale parameter, b—the shape parameter. (**C**) Alamar Blue test results after 1, 3, and 7 days of MG-63 cells culturing; indicates statistical significance when compared with: * ColChCSmod30_20 day 3; # HybC3 day 7. (**D**) Results of the alkaline phosphatase (ALP) activity test after 3 and 7 days of culturing MG-63 cells on the surface of materials; indicates statistical significance when compared with: * control day 3; # control day 7. (**E**) Alamar Blue test results after 1, 3 and 7 days of J774A.1 cells culturing; * indicates statistical significance when compared with ColChCS_mod_30_20 day 7. For (**D**,**E**), the black line indicates statistical significance between the results for the same type of material on the 3rd and 7th day. (**F**) The viability evaluation of bacterial colonies (*S. aureus* and *E. coli*) grown after contact with developed materials expressed as a percentage of positive control.

To examine the in vitro release of ALN, MSP-NH_2_-HAp-ALN particles were suspended in a PBS medium and incubated at 37 °C with gentle shaking. After the selected time frames, samples were centrifuged, and a UV-Vis spectra of resulting supernatants were collected. As alendronate does not exhibit a spectrum in the ultraviolet range, before measurement, the complexation with Fe^3+^ ions was conducted [55]. As can be seen in Figure 5A, depicting the cumulative mass of alendronate released during the experiment, the particles display a clear initial “burst effect”. During the first 24 h, about 70% of the total drug amount was released. However, the ALN was released for 10 days, rendering the entire alendronate content in MSP-NH_2_-HAp-ALN particles as 10%. The high BET surface and presence of pores allowed for higher drug loading compared to silica structures without pores and a low BET surface [18,42]. We postulate that during the first phase of the experiment, the alendronate was released from the pores of mesoporous silica, which contributed to the accelerated loss of the drug. The second phase could be related to the release of ALN, which is more strongly connected to hydroxyapatite, as this drug is reported to have a high affinity to this mineral phase [56,57]. Nevertheless, to limit this initial ‘burst release’ MSP-NH_2_-HAp-ALN particles were incorporated into hydrogel matrices; as such, the designed systems are able to slow down the release of the drugs [18,58,59,60]. Based on the presented results, expressed as the percentage of total ALN released from MSP-NH_2_-HAp-ALN particles from the hybrid system (Figure 5A), a significant decrease in the initial burst effect could be seen. After 24 h of the experiment, approximately 43% of total ALN content was released, which is more than 1.5 lower than the value for MSP-NH_2_-HAp-ALN after the corresponding time. After the 10 days of the experiment, all of the drug was released from particles; however, when these particles were suspended in a hydrogel matrix, the release lasted a minimum of 20 days, and after this time, the released amount of drug was close to 83%. This prolonged release and limited burst release effect from the first day makes the hybrid system superior as a drug delivery system compared to pure MSP-NH_2_-HAp-ALN particles. Additionally, the obtained data for the hybrid system were fitted to mathematical models—Higuchi, Weibull, and Ritger–Peppas [61,62,63] (Figure 5B). For the Higuchi and Ritger–Peppas models, 60% of data were used (the first 48 h). The Higuchi model is the simplest of all those presented, and it has the lowest correlation coefficient. The main drawbacks are the multiple hypotheses on which this model is based—negligible matrix dissolution and swelling, one-dimension drug diffusion, the initial drug concentration is higher than drug solubility, etc. According to the literature [63], a better model for the polymeric system is Ritger–Peppas. As the calculated n factor in the Ritger–Peppas model is lower than 0.45, the release in this time frame follows Fickian diffusion mechanism (n < 0.45—Fickian diffusion; 0.45 < n < 0.89—non-Fickian transport; n = 0.89—case II transport; n > 0.89—super case II transport [62]). From all of the presented models, the highest degree of the correlation coefficient was achieved for the Weibull model (R^2^ = 0.9689). The value of the b parameter equal to 0.4043 allows it to be further proved that, in the hybrid system, the drug release follows Fickian diffusion ((b < 0.75—Fickian diffusion; 0.75 < b < 1—combined mechanism, b = 1—first ordered release; b > 1—complex mechanism [64]).

### 2.6. Biological Evaluation of Composites in Osteoblast-like and Osteoclast-like Cell Culture In Vitro

In view of the expected biological role of tested composites, the preliminary studies on the biocompatibility in vitro utilizing MG-63 cells as an osteoblast-like model were performed. We have quantitatively evaluated the ability of developed materials to support cells proliferation, and additionally we assessed the alkaline phosphatase activity (ALP). The results of the Alamar Blue test on the 1st, 3rd, and 7th culture day are shown in Figure 5C. The analogous tendency for all tested materials was revealed, namely the number of metabolically active cells noticeably grew up after 3 days and decreased at day 7. After 3 days of culturing, the cells number is higher for composites when compared to pure hydrogel; however, the statistical significance was revealed only for HybC2. Conversely, on the 7th day of the experiment, the highest result was observed for HybC3 (statistical significance when compared to ColChCS_mod_ and HybC1). Thus, we found that the addition of inorganic particles loaded with ALN at the tested concentration (0.5, 1.25, 2.5 mg/1 mL of sol) does not deteriorate the biocompatibility of the resulting composites with respect to the plain hydrogel. We suggest that the lowest number of MG-63 cells cultured on developed materials surfaces might be related to the ability of those materials to promote differentiation rather than proliferation. It was reported that when cells start to differentiate, their proliferation decreases [65]. In order to verify this assumption and gain more insight into the functions of MG-63 cells cultured on the developed materials, we have evaluated the alkaline phosphate activity. ALP serves as one of the markers confirming the early differentiation of osteoblast-like cells and its expression changes with osteoblast activity [66,67]. To establish whether differentiation of studied cells is occurring when cultured on the surface of hybrid and hydrogel systems, the ALP activity was tested on the 3rd and 7th days of the experiment. The results of ALP activity measurements are illustrated in Figure 5D. The level of ALP increased after 7 days of culturing for all tested materials. Comparing ALP results for the same type of material, on the 3rd and the 7th days, the statistical significances were revealed in all cases. Moreover, it was found that ALP activity of cells cultured on studied materials was substantially higher compared to the cells on the tissue culture plate (TCP) at both experimental points (statistical significance). We observed a similar tendency in our previous work [68]. Furthermore, analyzing the impact of MSP-NH_2_-HAp-ALN particles concentration on ALP activity after 7 days of the experiment, no significant differences were revealed. We noticed that the addition of particles with ALN loaded/attached to the polymeric matrix slightly enhanced ALP activity (for HybC1 and HybC2) compared to the pure hydrogel (no statistical significance). The highest ALP activity was observed for HybC1 material on day 7 of the experiment. Overall, the biocompatibility of the prepared composites, as well as the ability to support ALP activity secreted by MG-63 cells cultured on their surface, were clearly established.

To establish the therapeutic potential of developed composites, a preliminary in vitro study on osteoclasts-like cells, J774A.1, was also carried out. The main features of J774A.1 cells are similar to osteoclasts, and therefore they serve as a reference cell line for evaluation of the bone resorption inhibition [69]. The results of the Alamar Blue test are depicted in Figure 5E. As can be seen, the number of cells cultured on the surface of all tested materials increased from the 1st to the 3rd day of the experiment, and subsequently decreased after 7 days of culturing. Importantly, for composites, this effect is clearly pronounced, with statistical significance revealed. Conversely, no significant differences were found when compared with the results for pristine hydrogels after 3 and 7 days of the experiment. The preliminary biological studies demonstrated that the proliferation of osteoclast-like cells cultured after 7 days on the composites are inhibited with respect to the control ColChCS_mod_ sample (statistical significance for HybC1 and HybC2). However, the particle content-dependent inhibition activity was not observed in the tested range of MSP-NH_2_-HAp-ALN concentration. Overall, it was proved that the composites developed herein substantially hampered the proliferation of J774A.1 cells, which in turn proves their therapeutic potential as materials for the treatment of osteoporosis. In particular, it should be emphasized that this effect was not as pronounced for our previous systems, for which only tendency was demonstrated [18]. We have confirmed that alendronate affects the bone cells while being introduced in the proposed formulations. Overall, it might be expected that the developed injectable composite-like ALN delivery system will be able to induce the local drug action, thus improving the effectiveness of anti-osteoporosis therapy.

### 2.7. Antibacterial Activity of Materials Developed

To demonstrate the multifunctional potential of developed hybrids, the antibacterial activity in vitro against *Staphylococcus aureus* (Gram-positive) and *Escherichia coli* (Gram-negative) was assessed. In Figure 5F, the results of antimicrobial tests against both types of bacteria strains are depicted. The number of bacterial colonies grown after contact with materials tested was expressed as a percentage of colonies on the positive control (plate). Chitosan is a polysaccharide well known for its antibacterial features [70]. In our previous work, we demonstrated the chitosan content-dependent intrinsic antibacterial activity of the chemically crosslinked hydrogels composed of collagen, hyaluronic acid, and chitosan in the in vitro experiment [71]. Herein, pristine chitosan-based hydrogel crosslinked with 20 mM of genipin solution was treated as a negative control and its high antibacterial activity was confirmed. Both types of bacterial strains were effectively killed after contact with this hydrogel, and the resulting percentage of the positive control reached about 0.015 and 0.005% for *S. aureus* and *E. coli,* respectively. For all developed materials containing 20 wt% of chitosan, similar antibacterial activity against both bacterial strains was revealed. The growth of bacteria was substantially inhibited when cultured on ColChCS_mod_ and hybrids systems. The obtained percentage of positive control for tested materials was in the range of 1.5–2.0% and 0.5–3.0% for *S. aureus* and *E. coli,* respectively. The highest number of bacterial colonies was observed for the pristine ColChCS_mod_ hydrogel, while the lowest was for the HybC3 system. It was noticed that, with an addition of MSP-NH_2_-HAp-ALN, the antimicrobial activity slightly increases compared to the pure hydrogel; however, the particles’ concentration-dependent activity was not found (no statistical significance). Son et al. [72], studied the antibacterial activity of mesoporous silica particles (MSNs) against *Escherichia coli* in vitro. They showed that MSNs toxicity was strongly influenced by the residual CTAB used in particle synthesis and not completely removed by an acid etching process. The surfactant-contaminated particles can induce the lysis of the cell membrane, thereby leading to the death of the bacteria. We have not observed the significant particle-content dependent changes in the viability of the bacteria which should be noticeable during such contamination. Moreover the osteoblast-like and osteoclast-like cells in vitro culturing demonstrated that the addition of developed silica-based particles does not deteriorate the biocompatibility of the composites in respect to the plain hydrogel. Taking into account the purification protocol utilized for MSP-NH_2_, as well as the results of in vitro evaluation, it might be expected that the key role in antimicrobial activity of the resulting composites plays the chitosan presence. In conclusion, the revealed antimicrobial activity of the developed systems against *Staphylococcus aureus* and *Escherichia coli* is further proof of their multifunctionality, and makes them very promising in biomedical applications.

## 3. Materials and Methods

Details of the materials used in this study are presented Supporting Materials (see the *Materials* section).

### 3.1. Synthesis of Functionalized Mesoporous Silica Particles with Amino Groups Decorated with Hydroxyapatite and Loaded/Attached with Alendronate (MSP-NH_2_-HAp-ALN)

The preparation of MSP-NH_2_-HAp-ALN was performed in three steps. First, 0.2 g of CTAB was dissolved in 100 mL of distilled water and 1.4 mL of 1 M NaOH was added. The solution was stirred for 2 h (80 °C, 500 rpm). Then, 2 mL of TEOS and 0.2 mL of APTES were added dropwise, and the stirring was continued under the same conditions for another 2 h. The resulting material was cooled down and centrifuged (5 °C, 9000 rpm, 10 min). The precipitate was washed twice with water and three times with 96% EtOH. Then, 2 g of NH_4_NO_3_ was dissolved in 200 mL of 96% ethanol and added to the obtained precipitate to remove surfactant particles. The resulting suspension was stirred (500 rpm) at 80 °C for 18 h. The material (MSP-NH_2_) was then centrifuged and rinsed three times with 96% EtOH and three times with water, frozen, and lyophilized for 24 h. In the second step, deposition of hydroxyapatite (HAp) on the surface of MSP-NH_2_ particles was performed under simulated body fluid (1.5 SBF) [53]. For that purpose, 20 mg of MSP-NH_2_ was suspended in 20 mL of 1.5× SBF by placing it in an ultrasonic bath for 5 min. The resulting suspension was then incubated at 37 °C with gentle shaking (50 rpm). The incubation was carried out for 5 days, with the material centrifuged after each 24 h (5 °C, 10,000 rpm, 20 min) and a fresh aliquot of 1.5× SBF was added. After the decoration was completed, the resulting material (MSP-NH_2_-HAp) was washed 3× with distilled water, frozen, and lyophilized. In the last step, 20 mg of MSP-NH_2_-HAp was suspended in 3 mL of a 5 mM NaOH solution by placing it in an ultrasonic bath for 5 min. Then, 20 mg of sodium alendronate was dissolved in 10 mL of 5 mM NaOH, and the pH was increased to 10 with 20 mM NaOH. The alendronate solution was added to the silica-containing suspension and stirred (500 rpm) at 37 °C for 72 h. After 3 days, the product (MSP-NH_2_-HAp-ALN) was centrifuged (5 °C, 10,000 rpm, 20 min), washed with deionized water, frozen, and lyophilized.

### 3.2. Model In Vitro Release of ALN from MSP-NH_2_-HAp-ALN

Next, 5 mg of the MSP-NH_2_-HAp-ALN sample were suspended in 1.5 mL of PBS buffer and placed in an incubator with gentle shaking (37 °C, 50 rpm). After the selected time frames (0.5 h, 1 h, 2 h, 4 h, 8 h, 24 h, 48 h, 96 h, 10 days), the suspension was centrifuged (10,000 rpm, 20 min, 5 °C), and the solution was removed for further measurements and a fresh portion of buffer was added. To quantify the amount of the released ALN, to 0.5 mL of the obtained solution, 2.35 mL of 0.2 M HClO_4_ and 0.15 mL of 5 mM FeCl_3_ in 0.2 M HClO_4_ were added. UV-Vis measurements performed on a UV-Vis spectrophotometer (Varian Cary 50) were conducted after 5 min, and absorbance at 300 nm was read [18]. Samples without Fe^3+^ ions were used as a blank. Experiments were performed in triplicates.

### 3.3. Characterization of MSP-NH_2_, MSP-NH_2_-HAp, and MSP-NH_2_-HAp-ALN Particles

SEM imaging was conducted with the use of the cold field emission scanning electron microscope (SEM) HITACHI S-4700. The suspension of the sample was drop-casted onto the silicon wafer, which was cleaned with piranha solution and rinsed with deionized water. Before measurement, the samples were sputtered with gold. TEM imaging was performed with FEI Tecnai Osiris equipment. XPS (X-ray photoelectron spectroscopy) analyses were conducted with a multifunctional ESCA instrument. The obtained results were analyzed with the CasaXPS program(Version 2.3.24). FTIR spectra were recorded using a Nicolet iS10FT-IR spectrometer equipped with an ATR accessory (SMART iTX). Thermal decomposition of materials was studied using a Mettler-Toledo TGA/SDTA851e thermogravimeter. The XRD patterns were recorded in Bragg–Brentano configuration with an X’Pert Pro Philips diffractometer. The specific surface area (S_BET_) and pore size distribution were determined from the nitrogen adsorption isotherms obtained at −196 °C using a 3Flex (Micromeritics, Norcross, GA, USA) automated gas adsorption system. Before the measurements, the samples were degassed under 0.2 mbar at 100 °C for 24 h. The pore size was evaluated from the adsorption branch of nitrogen isotherm using the Barrett–Joyner–Halenda (BJH) equation with the Kruk–Jaroniec–Sayari (KJS) correction. The pore volume was calculated based on the total amount of nitrogen adsorbed at p/p_0_ = 0.98.

### 3.4. Hybrid Preparation

The hybrid composed of 50 wt% collagen, 20 wt% chitosan, and 30 wt% lysine functionalized chondroitin sulfate with 0.5 mg, 1.25 mg, 2.5 mg of MSP-NH_2_-HAp-ALN per 1 mL of sol denoted as HybC1, HybC2, HybC3 were prepared by mixing 259 µL of the stock collagen solution with 42 µL of 1 wt% chitosan solution in 1% acetic acid and 63 µL of 1 wt% modified chondroitin sulfate solution in 10× PBS buffer. In the next step, 50 µL suspension of the selected concentration of particles and 85 µL of 20 mM genipin solution in 10× PBS buffer were added. The obtained polymeric sols were vortexed and placed in an incubator at 37 °C until gel formation. The pure ColChCS_mod_30_20 hydrogel was prepared analogously, except that instead of particles suspension, 50 µL of water was added to polymeric sol. The crosslinking time evaluated by the inverted vial test was in the range of 5 min for all samples studied. However, systems were incubated at 37 °C for 24 h before further evaluation, and gels changed color from milky white to dark greyish-blue cream, characteristic of the genipin-involved crosslinking.

### 3.5. Physicochemical Characterization of Hybrids

**For degradability**, hybrids were placed in a 24-well plate, and 1 mL of collagenase solution type I (0.2 mg/mL, 1 mL, ≥125 U/mg) in 1× PBS with 0.36 mM CaCl_2_ was added; then, the materials were incubated at 37 °C with gentle shaking (50 rpm). At the given time points (4 h, 24 h, 48 h, 72 h, 144 h), the samples were weighed and a fresh portion of the enzyme was added each time. For each sample, the experiments of degradation were carried out in triplicate and the results are presented as averages. **The swelling** of the hybrids was tested under physiological conditions by incubating the materials at 37 °C in PBS buffer with gentle shaking (50 rpm) for 24 h. After this time, the buffer was collected and the samples were washed twice with deionized water and weighed (W_s_). The materials were frozen, lyophilized, and reweighed (W_d_), and the swelling ratio (SR) was calculated using the formula:

(1) $$\mathrm{SR}=\frac{W_{s}-W_{d}}{W_{d}}\cdot 100\%.$$

For each type of hybrid, the experiment was carried out in triplicate and the result is presented as the mean. **The wettability** of hydrogels was analyzed by contact angle measurements. Five contact angle values were measured for each sample using the ImageJ program with the contact angle plugin, and the average value was calculated. **Rheological measurement** was conducted with an MCR 301 Anton Paar rotational rheometer with a P-PTD200 measurement cell and an H-PTD200 chamber to maintain high-temperature stabilization during experiments. Amplitude sweep (AS) tests were carried out using plate–plate measurement geometry, plate diameter d = 25 mm, gap height h = 0.1 mm, and temperature T = 37 °C. The samples crosslinked beforehand were prepared as described above, and transferred to the rheometer. Then, 0.5 mL of hydrogel was placed on the bottom plate (plate temperature 37 °C) and the top plate was gradually lowered to 2, 1.5, 1, 0.8, 0.6, and 0.5 mm. After the assessment of the filling of the measuring gap, the AS tests lasting 7–8 min were performed for γ = 0.01–100%, f = 1 Hz. In the next step, the height of the measuring gap was set as 0.1 mm. Proper second AS tests were conducted with the parameters from the first measurement. Each sample was prepared in triplicate, and the results are presented as the mean.

### 3.6. Drug Release Studies

For experimental details on drug release studies from developed composites, please visit the Supporting Materials.

### 3.7. In Vitro Biomineralization

In vitro biomineralization studies were performed in simulated body fluid (SBF) prepared according to Kokubo’s method [53]. For more details, please see the Supporting Materials.

### 3.8. Biological Experiments In Vitro Employing Osteoblast-like (MG-63) and Osteoclast-like (J774A.1) Cells Culture

For experimental details on in vitro tests, please visit the Supporting Materials.

### 3.9. In Vitro Antibacterial Activity Studies

For experimental details on in vitro antibacterial tests, please visit the Supporting Materials.

### 3.10. Statistical Analysis

Experiments were repeated three times, and results were expressed as a mean ± standard deviation. Statistical significance was calculated using the Student’s *t*-test. A comparison between the two means was analyzed with a statistical significance level set at *p* < 0.05.

## 4. Conclusions

The novel composites based on collagen/chitosan/chondroitin sulfate hydrogel reinforced with amino-functionalized mesoporous silica particles decorated with hydroxyapatite and loaded with alendronate (MSP-NH_2_-HAp-ALN) were designed and characterized as a potential, injectable material for osteoporosis treatment. MSP-NH_2_ particles (S_BET_ = 613 m^2^/g, d_por_ = 3.7 nm, V_por_ = 0.57 cm^3^/g) were effectively decorated with hydroxyapatite under biomimetic conditions (5 days of incubation in 1.5× SBF), which was confirmed using XRD, SEM, and EDS measurements. The particles were loaded with ALN, with the drug content established as 10% based on UV-Vis spectroscopy and 14% based on thermogravimetry. Hydrogel-based composites with three concentrations of MSP-NH_2_-HAp-ALN (HybC1, HybC2, HybC3) were fabricated and characterized in terms of physicochemical and biological features. It was found that the addition of particles caused a decrease in wettability and swellability, and these effects were more pronounced in systems with higher particle concentration. The involvement of MSP-NH_2_-HAp-ALN in the crosslinking process was established, as well as the ability of hybrids to be used as injectable systems. The incorporation of inorganic particles into the hydrogel matrix improved its mechanical properties and the effect was related to the MSP-NH_2_-HAp-ALN concentration. Bioactivity study revealed that the formation of mineral phases is more pronounced in HybC2 and HybC3. Importantly, the prolonged release (up to 20 days) and limited burst release effect from the first day makes the hybrid system superior as the drug delivery system compared to pure MSP-NH_2_-HAp-ALN particles. Biocompatibility of prepared composites was established in vitro with Alamar Blue and ALP activity tests performed for osteoblast-like cells. Finally, the observed hampering of model osteoclast-like cell proliferation, while cultured on the surface of developed composites, proved their therapeutic potential as the materials for the treatment of osteoporosis. Furthermore, the antibacterial activity of the composites in in vitro experiments was also demonstrated. Overall, the novel multifunctional delivery systems presented within this work seem to represent an extremely promising alternative to the formulation investigated so far. The developed materials simultaneously possess antiosteoporotic and antimicrobial properties, and mimic the architecture and chemical composition of the natural bone tissue, thus supporting bone regeneration and biointegration while displaying structural stability and desired physicochemical features. However, to prove their usefulness in bone tissue engineering, the biological evaluation in vivo is still required.

## Data Availability

The data presented in this study are available on request from the corresponding author.

## Supplementary Materials

The following supporting information can be downloaded at: https://www.mdpi.com/article/10.3390/ijms24054959/s1. Materials and Methods in supplementary materials present Drug release studies from developed composites, In vitro biomineralization, Osteo-blast-like (MG-63) and osteoclast-like (J774A.1) cells culture, Alamar Blue and Alkaline phosphatase (ALP) assays, In vitro antibacterial activity studies. References [20,53,55,65,71,73] are cited in supplementary materials.

## References

[1] Carlson B.M. (2019). The Skeleton. Hum. Body.

[2] Gisbert-Garzarán M., Manzano M., Vallet-Regí M. (2020). Mesoporous Silica Nanoparticles for the Treatment of Complex Bone Diseases: Bone Cancer, Bone Infection and Osteoporosis. Pharmaceutics.

[3] Li D., Zhou J., Zhang M., Ma Y., Yang Y., Han X., Wang X. (2020). Long-Term Delivery of Alendronate through an Injectable Tetra-PEG Hydrogel to Promote Osteoporosis Therapy. Biomater. Sci..

[4] Kanis J.A., Norton N., Harvey N.C., Jacobson T., Johansson H., Lorentzon M., McCloskey E.V., Willers C., Borgström F. (2021). SCOPE 2021: A New Scorecard for Osteoporosis in Europe. Arch. Osteoporos..

[5] Furman B.L. (2016). Alendronate. Reference Module in Biomedical Sciences.

[6] Khan M., Cheung A.M., Khan A.A. (2017). Drug-Related Adverse Events of Osteoporosis Therapy. Endocrinol. Metab. Clin. N. Am..

[7] Kuźnik A., Październiok-Holewa A., Jewula P., Kuźnik N. (2020). Bisphosphonates—Much More than Only Drugs for Bone Diseases. Eur. J. Pharmacol..

[8] Eastell R. (2013). Osteoporosis. Medicine.

[9] Tu K.N., Lie J.D., Wan C.K.V., Cameron M., Austel A.G., Nguyen J.K., Van K., Hyun D. (2018). Osteoporosis: A Review of Treatment Options. Pharm. Ther..

[10] Nafee N., Zewail M., Boraie N. (2018). Alendronate-Loaded, Biodegradable Smart Hydrogel: A Promising Injectable Depot Formulation for Osteoporosis. J. Drug Target..

[11] Horikawa A., Miyakoshi N., Shimada Y., Sugimura Y., Kodama H. (2015). A Comparative Study between Intravenous and Oral Alendronate Administration for the Treatment of Osteoporosis. SpringerPlus.

[12] Klara J., Lewandowska-Łańcucka J. (2022). How Efficient Are Alendronate-Nano/Biomaterial Combinations for Anti-Osteoporosis Therapy? An Evidence-Based Review of the Literature. Int. J. Nanomed..

[13] Dong J., Tao L., Abourehab M.A.S., Hussain Z. (2018). Design and Development of Novel Hyaluronate-Modified Nanoparticles for Combo-Delivery of Curcumin and Alendronate: Fabrication, Characterization, and Cellular and Molecular Evidences of Enhanced Bone Regeneration. Int. J. Biol. Macromol..

[14] Dolci L.S., Panzavolta S., Albertini B., Campisi B., Gandolfi M., Bigi A., Passerini N. (2018). Spray-Congealed Solid Lipid Microparticles as a New Tool for the Controlled Release of Bisphosphonates from a Calcium Phosphate Bone Cement. Eur. J. Pharm. Biopharm..

[15] Wu H., Lei P., Liu G., Shrike Zhang Y., Yang J., Zhang L., Xie J., Niu W., Liu H., Ruan J. (2017). Reconstruction of Large-Scale Defects with a Novel Hybrid Scaffold Made from Poly(L-lactic acid)/nanohydroxyapatite/Alendronate-Loaded Chitosan Microsphere: In Vitro and in Vivo Studies. Sci. Rep..

[16] Yuan W., Li Z., Xie X., Zhang Z.-Y., Bian L. (2020). Bisphosphonate-Based Nanocomposite Hydrogels for Biomedical Applications. Bioact. Mater..

[17] Posadowska U., Parizek M., Filova E., Wlodarczyk-Biegun M., Kamperman M., Bacakova L., Pamula E. (2015). Injectable Nanoparticle-Loaded Hydrogel System for Local Delivery of Sodium Alendronate. Int. J. Pharm..

[18] Gilarska A., Hinz A., Bzowska M., Dyduch G., Kamiński K., Nowakowska M., Lewandowska-Łańcucka J. (2021). Addressing the Osteoporosis Problem—Multifunctional Injectable Hybrid Materials for Controlling Local Bone Tissue Remodeling. ACS Appl. Mater. Interfaces.

[19] Narayan R., Nayak U., Raichur A., Garg S. (2018). Mesoporous Silica Nanoparticles: A Comprehensive Review on Synthesis and Recent Advances. Pharmaceutics.

[20] Klara J., Marczak A., Łatkiewicz A., Horak W., Lewandowska-Łańcucka J. (2022). Lysine-Functionalized Chondroitin Sulfate Improves the Biological Properties of Collagen/Chitosan-Based Injectable Hydrogels. Int. J. Biol. Macromol..

[21] Shirazi S., Ravindran S., Cooper L.F. (2022). Topography-Mediated Immunomodulation in Osseointegration; Ally or Enemy. Biomaterials.

[22] Chen R., Hao Z., Wang Y., Zhu H., Hu Y., Chen T., Zhang P., Li J. (2022). Mesenchymal Stem Cell–Immune Cell Interaction and Related Modulations for Bone Tissue Engineering. Stem Cells Int..

[23] Manzano M., Colilla M., Vallet-Reg M. (2009). Drug Delivery from Ordered Mesoporous Matrices. Expert Opin. Drug Deliv..

[24] Newham G., Evans S.D., Ong Z.Y. (2022). Mechanically Tuneable Physical Nanocomposite Hydrogels from Polyelectrolyte Complex Templated Silica Nanoparticles for Anionic Therapeutic Delivery. J. Colloid Interface Sci..

[25] Wu S.-H., Mou C.-Y., Lin H.-P. (2013). Synthesis of Mesoporous Silica Nanoparticles. Chem. Soc. Rev..

[26] Thommes M., Kaneko K., Neimark A.V., Olivier J.P., Rodriguez-Reinoso F., Rouquerol J., Sing K.S.W. (2015). Physisorption of Gases, with Special Reference to the Evaluation of Surface Area and Pore Size Distribution (IUPAC Technical Report). Pure Appl. Chem..

[27] Cho S.B., Miyaji F., Kokubo T., Nakanishi K., Soga N., Nakamura T. (1996). Apatite Formation on Various Silica Gels in a Simulated Body Fluid Containing Excessive Calcium Ion. J. Ceram. Soc. Japan.

[28] Hamai R., Shirosaki Y., Miyazaki T. (2018). Structural Effects of Sulfur-Containing Functional Groups on Apatite Formation on Ca2+-Modified Copolymers in a Simulated Body Environment. ACS Omega.

[29] Miyazaki T., Ohtsuki C. (2008). Design of Bioactive Bone Cement Based on Organic–Inorganic Hybrids. Orthopaedic Bone Cements.

[30] Alves N.M., Leonor I.B., Azevedo H.S., Reis R.L., Mano J.F. (2010). Designing Biomaterials Based on Biomineralization of Bone. J. Mater. Chem..

[31] Albayati T.M., Salih I.K., Alazzawi H.F. (2019). Synthesis and Characterization of a Modified Surface of SBA-15 Mesoporous Silica for a Chloramphenicol Drug Delivery System. Heliyon.

[32] Kotian R., Rao P.P., Madhyastha P. (2017). X-Ray Diffraction Analysis of Hydroxyapatite-Coated in Different Plasma Gas Atmosphere on Ti and Ti-6Al-4V. Eur. J. Dent..

[33] Rogina A., Šandrk N., Teruel-Biosca L., Antunović M., Ivanković M., Ferrer G.G. (2020). Bone-Mimicking Injectable Gelatine/Hydroxyapatite Hydrogels. Chem. Biochem. Eng. Q..

[34] Wei S., Ma J.X., Xu L., Gu X.S., Ma X.L. (2020). Biodegradable Materials for Bone Defect Repair. Mil. Med. Res..

[35] Velasco M.A., Narváez-Tovar C.A., Garzón-Alvarado D.A. (2015). Design, Materials, and Mechanobiology of Biodegradable Scaffolds for Bone Tissue Engineering. BioMed Res. Int..

[36] MacAya D., Ng K.K., Spector M. (2011). Injectable Collagen–Genipin Gel for the Treatment of Spinal Cord Injury: In Vitro Studies. Adv. Funct. Mater..

[37] Guo X., Park H., Temenoff J.S., Tabata Y., Caplan A.I., Mikos A.G. (2008). Effect of Swelling Ratio of Injectable Hydrogel Composites on Chondrogenic Differentiation of Encapsulated Rabbit Marrow Mesenchymal Stem Cells in Vitro. AIChE Annu. Meet. Conf. Proc..

[38] Ikeda T., Ikeda K., Yamamoto K., Ishizaki H., Yoshizawa Y., Yanagiguchi K., Yamada S., Hayashi Y. (2014). Fabrication and Characteristics of Chitosan Sponge as a Tissue Engineering Scaffold. Biomed Res. Int..

[39] Murphy C.M., Haugh M.G., O’Brien F.J. (2010). The Effect of Mean Pore Size on Cell Attachment, Proliferation and Migration in Collagen-Glycosaminoglycan Scaffolds for Bone Tissue Engineering. Biomaterials.

[40] Karoyo A.H., Wilson L.D. (2021). A Review on the Design and Hydration Properties of Natural Polymer-Based Hydrogels. Materials.

[41] Patel A., Mequanint K. (2011). Hydrogel Biomaterials. Biomedical Engineering—Frontiers and Challenges.

[42] Lewandowska-Łańcucka J., Gilarska A., Buła A., Horak W., Łatkiewicz A., Nowakowska M. (2019). Genipin Crosslinked Bioactive Collagen/Chitosan/Hyaluronic Acid Injectable Hydrogels Structurally Amended via Covalent Attachment of Surface-Modified Silica Particles. Int. J. Biol. Macromol..

[43] Zengin A., Castro J.P.O., Habibovic P., Van Rijt S.H. (2021). Injectable, Self-Healing Mesoporous Silica Nanocomposite Hydrogels with Improved Mechanical Properties. Nanoscale.

[44] Gaharwar A.K., Rivera C., Wu C.J., Chan B.K., Schmidt G. (2013). Photocrosslinked Nanocomposite Hydrogels from PEG and Silica Nanospheres: Structural, Mechanical and Cell Adhesion Characteristics. Mater. Sci. Eng. C.

[45] Arcos D., Boccaccini A.R., Bohner M., Díez-Pérez A., Epple M., Gómez-Barrena E., Herrera A., Planell J.A., Rodríguez-Mañas L., Vallet-Regí M. (2014). The Relevance of Biomaterials to the Prevention and Treatment of Osteoporosis. Acta Biomater..

[46] Gittens R.A., Scheideler L., Rupp F., Hyzy S.L., Geis-Gerstorfer J., Schwartz Z., Boyan B.D. (2014). A Review on the Wettability of Dental Implant Surfaces II: Biological and Clinical Aspects. Acta Biomater..

[47] Siqueira R., Ferreira J.A., Rizzante F.A.P., Moura G.F., Mendonça D.B.S., de Magalhães D., Cimões R., Mendonça G. (2021). Hydrophilic Titanium Surface Modulates Early Stages of Osseointegration in Osteoporosis. J. Periodontal Res..

[48] Calciolari E., Hamlet S., Ivanovski S., Donos N. (2018). Pro-Osteogenic Properties of Hydrophilic and Hydrophobic Titanium Surfaces: Crosstalk between Signalling Pathways in in Vivo Models. J. Periodontal Res..

[49] Boyan B.D., Lotz E.M., Schwartz Z. (2017). Roughness and Hydrophilicity as Osteogenic Biomimetic Surface Properties. Tissue Eng.—Part A.

[50] Zimina A., Senatov F., Choudhary R., Kolesnikov E., Anisimova N., Kiselevskiy M., Orlova P., Strukova N., Generalova M., Manskikh V. (2020). Biocompatibility and Physico-Chemical Properties of Highly Porous PLA/HA Scaffolds for Bone Reconstruction. Polymers.

[51] Liu L., Li X., Shi X., Wang Y. (2018). Injectable Alendronate-Functionalized GelMA Hydrogels for Mineralization and Osteogenesis. RSC Adv..

[52] Yan C., Pochan D.J. (2010). Rheological Properties of Peptide-Based Hydrogels for Biomedical and Other Applications. Chem. Soc. Rev..

[53] Kokubo T., Takadama H. (2006). How Useful Is SBF in Predicting in Vivo Bone Bioactivity?. Biomaterials.

[54] Sobczak-Kupiec A., Drabczyk A., Florkiewicz W., Głąb M., Kudłacik-Kramarczyk S., Słota D., Tomala A., Tyliszczak B. (2021). Review of the Applications of Biomedical Compositions Containing Hydroxyapatite and Collagen Modified by Bioactive Components. Materials.

[55] Kuljanin J., Jankovi I., Nedeljkovi J., Prstojevi D., Marinkovi V. (2002). Spectrophotometric Determination of Alendronate in Pharmaceutical Formulations via Complex Formation with Fe(III) Ions. J. Pharm. Biomed. Anal..

[56] Yewle J.N., Puleo D.A., Bachas L.G. (2011). Enhanced Affinity Bifunctional Bisphosphonates for Targeted Delivery of Therapeutic Agents to Bone. Bioconjug. Chem..

[57] Capra P., Dorati R., Colonna C., Bruni G., Pavanetto F., Genta I., Conti B. (2011). A Preliminary Study on the Morphological and Release Properties of Hydroxyapatite-Alendronate Composite Materials. J. Microencapsul..

[58] Peers S., Montembault A., Ladavière C. (2020). Chitosan Hydrogels for Sustained Drug Delivery. J. Control. Release.

[59] Zhang H., Zhu Y., Qu L., Wu H., Kong H., Yang Z., Chen D., Mäkilä E., Salonen J., Santos H.A. (2018). Gold Nanorods Conjugated Porous Silicon Nanoparticles Encapsulated in Calcium Alginate Nano Hydrogels Using Microemulsion Templates. Nano Lett..

[60] Li J., Mooney D.J. (2016). Designing Hydrogels for Controlled Drug Delivery. Nat. Rev. Mater..

[61] Baishya H. (2017). Application of Mathematical Models in Drug Release Kinetics of Carbidopa and Levodopa ER Tablets. J. Dev. Drugs.

[62] Dash S., Murthy P.N., Nath L., Chowdhury P. (2010). Kinetic Modeling on Drug Release from Controlled Drug Delivery Systems. Acta Pol. Pharm.—Drug Res..

[63] Paarakh M.P., Jose P.A.N.I., Setty C.M., Peter G. (2019). V Release Kinetics—Concepts and Applications. Int. J. Pharm. Res. Technol..

[64] Papadopoulou V., Kosmidis K., Vlachou M., Macheras P. (2006). On the Use of the Weibull Function for the Discernment of Drug Release Mechanisms. Int. J. Pharm..

[65] Filipowska J., Lewandowska-Łańcucka J., Gilarska A., Niedźwiedzki Ł., Nowakowska M. (2018). In Vitro Osteogenic Potential of Collagen/Chitosan-Based Hydrogels-Silica Particles Hybrids in Human Bone Marrow-Derived Mesenchymal Stromal Cell Cultures. Int. J. Biol. Macromol..

[66] Nasello G., Alamán-Díez P., Schiavi J., Pérez M.Á., McNamara L., García-Aznar J.M. (2020). Primary Human Osteoblasts Cultured in a 3D Microenvironment Create a Unique Representative Model of Their Differentiation Into Osteocytes. Front. Bioeng. Biotechnol..

[67] Tsai S.W., Liou H.M., Lin C.J., Kuo K.L., Hung Y.S., Weng R.C., Hsu F.Y. (2012). MG63 Osteoblast-like Cells Exhibit Different Behavior When Grown on Electrospun Collagen Matrix versus Electrospun Gelatin Matrix. PLoS ONE.

[68] Krajcer A., Klara J., Horak W., Lewandowska-Łańcucka J. (2022). Bioactive Injectable Composites Based on Insulin-Functionalized Silica Particles Reinforced Polymeric Hydrogels for Potential Applications in Bone Tissue Engineering. J. Mater. Sci. Technol..

[69] Moreau M.F., Guillet C., Massin P., Chevalier S., Gascan H., Baslé M.F., Chappard D. (2007). Comparative Effects of Five Bisphosphonates on Apoptosis of Macrophage Cells in Vitro. Biochem. Pharmacol..

[70] Michalska-Sionkowska M., Kaczmarek B., Walczak M., Sionkowska A. (2018). Antimicrobial Activity of New Materials Based on the Blends of Collagen/Chitosan/Hyaluronic Acid with Gentamicin Sulfate Addition. Mater. Sci. Eng. C.

[71] Gilarska A., Lewandowska-Łańcucka J., Guzdek-Zając K., Karewicz A., Horak W., Lach R., Wójcik K., Nowakowska M. (2020). Bioactive yet Antimicrobial Structurally Stable Collagen/Chitosan/Lysine Functionalized Hyaluronic Acid-Based Injectable Hydrogels for Potential Bone Tissue Engineering Applications. Int. J. Biol. Macromol..

[72] Son M.J., Lee S.-W. (2021). Antibacterial Toxicity of Mesoporous Silica Nanoparticles with Functional Decoration of Specific Organic Moieties. Colloids Surf. A Physicochem. Eng. Asp..

[73] Gilarska A., Lewandowska-Łańcucka J., Horak W., Nowakowska M. (2018). Collagen/Chitosan/Hyaluronic Acid-Based Injectable Hydrogels for Tissue Engineering Applications—Design, Physicochemical and Biological Characterization. Colloids Surf. B Biointerfaces.

