# Supplementary material for: Towards Controlling the Local Bone Tissue Remodeling—Multifunctional Injectable Composites for Osteoporosis Treatment
Source: Int J Mol Sci. 2023 Mar 4;24(5):4959. doi: 10.3390/ijms24054959 (PMC10002562; doi:10.3390/ijms24054959)
Supplement: Supplementary file 1 [file ijms-24-04959-s001.zip › ijms-2235192-supplementary.pdf]

**Supplementary materials**

# Towards controlling the local bone tissue remodeling – multifunctional injectable composites for osteoporosis treatment

Joanna Klara[1], Sylwia Onak[1], Andrzej Kowalczyk[1], Wojciech Horak[2], Kinga Wójcik[3], and Joanna Lewandowska-Łańcucka[1]*

[1]Faculty of Chemistry, Jagiellonian University, Gronostajowa 2, 30-387 Kraków, Poland

[2] Department of Machine Design and Technology, Faculty of Mechanical Engineering and Robotics, AGH University of Science and Technology , , Al. Mickiewicza 30, 30-059 Kraków, Poland

[3]Department of Microbiology, Faculty of Biochemistry, Biophysics and Biotechnology, Jagiellonian University, Gronostajowa 7, 30-387 Kraków, Poland

## Experimental section

### *Materials*

Cetyltrimethylammonium bromide (CTAB, Acros Organics, 99+%, Belgium), Tetraethoxysilane(TEOS, Sigma Aldrich ⩾ 99% GC, Germany), (3-aminopropyl)trietoxysilane (APTES, Sigma Aldrich, ⩾ 98%, Germany), Ammonium nitrate ($NH_4NO_3$, Eurochem BGD, Poland), Ethanol ($C_2H_5OH$, POCH, 96%, Poland), Sodium chloride (NaCl, Chempur, Poland), Sodium bicarbonate ($NaHCO_3$, POCH, Poland), Potassium chloride (KCl, POCH, Poland), Dipotassium phosphate trihydrate ($K_2HPO_4 \cdot 3H_2O$, Sigma Aldrich, ⩾ 99%, Germany), Magnesium chloride hexahydrate ($MgCl_2 \cdot 6H_2O$, POCH, Poland), Anhydrous calcium chloride ($CaCl_2$, Sigma Aldrich, 93%, Germany) Tris (hydroxymethyl) aminomethane (TRIS, Sigma Aldrich, ⩾ 99,8%, Germany), Hydrochloric acid (HCl, Chempur, 35-38%, Poland), Sodium hydroxide (NaOH, POCH, Poland), Sodium alendronate (ALN, Sigma-Aldrich, Germany), Perchloric acid ($HClO_4$, Sigma-Aldrich, Germany), Saline solution in phosphate buffer (PBS, tablet, Sigma-Aldrich, Germany), Iron (III) chloride hexahydrate ($FeCl_3 \cdot 6H_2O$, Sigma-Aldrich, Germanny), Collagen type I rat tail (4.06 mg/ml solution, BD Biosciences, USA-CA), chitosan (low molecular weight, Sigma-Aldrich; Mv = 120 kDa, 79% deacetylated, Germany), genipin (Challenge Bioproducts Co., 98%, Taiwan), acetic acid (Chempur, Poland), L-Lysine monohydrochloride (Lys, Sigma-Aldrich, ≥98%, Germany), 2-(N-morpholino)ethane-sulfonic acid (MES, Sigma Aldrich, ≥99%, Germany), N-(3-dimethylaminopropyl)-N′-ethylcarbodiimide hydrochloride (EDC, Sigma Aldrich, 99%,

Germany), N-hydroxysuccinimide (NHS, Sigma-Aldrich, 98%, Germany), sodium carbonate (anhydrous ≥ 99.5%, POCH, Poland), collagenase (≥125 U/mg, Sigma-Aldrich, Germany), Dulbecco's Modified Eagle Medium (DMEM, Sigma-Aldrich, Germany), penicylin-streptomycin solution (10.00 units/ml, HyClone, USA-MA), fetal bovine serum (FBS, HyClone), trypsin (HyClone, USA-MA), Alamar Blue reagent (Invitrogen, USA-MA), glutaraldehyde solution (Sigma-Aldrich, Germany), hexamethyldisilazane reagent grade (HMDS, Sigma-Aldrich, Germany), osteoblasts-like **cells: MG-63** (ATCC® CRL-1427™, USA-VA) (Organism: Homo sapiens; Tissue: bone; Disease: osteosarcoma) and **osteoclast-like cells: J774A.1** (ATCC® TIB-67™, USA-VA) (Organism: Mus musculus; Cell Type: macrophage; Disease: reticulum cell sarcoma) were from American Type Culture Collection.

Chondroitin sulfate A sodium salt from bovine trachea (Sigma-Aldrich, Mv = 22.9 kDa) was functionalized with lysine ($CS_{mod}$) according to the procedure described by us earlier [1] (substitution degree about 21% was calculated based on $^{1}$HNMR spectroscopy),

*Methods*

*Drug release studies from developed composites*

Hybrid materials with the highest concentration of MSP-$NH_2$-HAp-ALN particles (HybC3) were taken for drug release evaluation. For that purpose, samples were placed in a 24-well plate and 1.25ml of PBS buffer was added to each well and they were incubated at 37ºC with gentle shaking (50rpm). At certain time points (1h, 2h,4h,8h,24h,48h,96h, 8 days, 12 days, 20 days) all the release medium was collected and replaced with fresh PBS. The same procedure was used for drug-free hybrid materials that were used as the reference. To determine the amount of released drug the spectrophotometric method based on the formation of a complex of alendronate and Fe(III) ions was used.[2] For this purpose 0.5 ml of collected sample was mixed with 2.35ml of 0.2M $HClO_4$ and 0.15ml of 5mM $FeCl_3$ in 0.2M $HClO_4$. UV-Vis measurements were conducted after 5 minutes and absorbance at 300nm was read. Samples without $Fe^{3+}$ ions were used as a blank. The concentration of ALN was determined based on the calibration curve for ALN. For each sample, the experiments were performed in triplicates, and the results are presented as the averages.

*In vitro biomineralization*

*In vitro* biomineralization studies were performed in Simulated body fluid (SBF) prepared according to Kokubo's method [3]. Briefly, 1ml of SBF solution was added to each well in a 24-well plate in which hybrids materials were obtained. After 24h of incubation at 37ºC with

gentle shaking, the SBF solution was removed, and a fresh portion was added. The procedure was repeated every day during the experiment. After the selected time frame (3,5,7 days), materials were rinsed three times with distilled water, frozen, and lyophilized. Obtained dry hybrids were prepared for SEM and EDS measurements (SEM, HITACHI S-4700 equipped with a NORAN Vantage energy dispersion spectrometer) by sticking on carbon tape and coating with a thin layer of gold.

*Biological experiments in vitro*

*Osteoblast-like (MG-63) and osteoclast-like (J774A.1) cells culture*

MG-63 and J774A.1 cells were cultured in Dulbecco's Modified Eagle's Medium–high glucose with 4500 mg/L glucose, L-glutamine, sodium pyruvate, and sodium bicarbonate, liquid, sterile-filtered, suitable for cell culture (DMEM) supplemented with 10 % (v/v) fetal bovine serum (FBS) and 100 U/mL penicillin and 100 µg/mL streptomycin (HyClone) in a humidified atmosphere (90% humidity) with 5 % $CO_2$ at 37°C. Cells were cultured in the standard tissue culture flasks. The medium was changed three times weekly. Before seeding on the materials, MG-63 cells were washed twice with PBS solution and subsequently harvested after 3 min incubation with 1 mL of 0.25% trypsin with 0.1% EDTA. After adding 3 mL of DMEM (with 10% (v/v) FBS) the cell suspension was centrifuged at 1000 rpm for 5 min, the supernatant was removed, and the pellet was resuspended in the culture medium. For J774A.1 cells, subcultures were prepared by scraping. Next, the collected cell suspension was centrifuged and resuspended analogously as MG-63 cells.

*Alamar Blue and Alkaline phosphatase (ALP) assays*

For the biological tests, the series of developed materials were prepared in 96/24-well plate, sterilized with UV light for 20 min, washed twice with PBS and next filled with medium (without serum) and left for about 1h in the incubator (37°C, 5% $CO_2$). Before cell culture, the medium was removed, and cells were seeded in the plate at a density of about $2x10^4$ cells per $cm^2$. 90 vol% of DMEM (supplemented with 1 vol% of penicillin-streptomycin solution) and 10 vol% of serum was used as a medium. The cell viability was studied using Alamar Blue (AB) assay after 1$^{st}$, 3$^{rd}$ and 7$^{th}$ day of culture as mentioned in previous works[1,4]. Based on standard curves established with MG-63/ J774A.1 cells cultured at various densities the cells number was calculated. For each sample, the experiments were carried out in triplicates and the results are presented as the averages. To measure ALP activity, materials were prepared in 24-well plate, and MG-63 cells culturing were conducted as mentioned earlier. ALP activity was

studied at culture day 3 and 7 using the protocol described in[5]. The calculation was based on the assumption that 1 mol ALP hydrolyzes 1 mol of pNPP substrate and thus ALP activity was expressed as the amount (nmol) of the product (p-nitrophenyl, pNP).

*In vitro antibacterial activity studies*

The antibacterial activity of obtained materials against the *Staphylococcus aureus* (ATCC 6538) (Gram-positive bacteria) and *Escherichia coli* (ATCC 25922) (Gram-negative bacteria) was evaluated using the protocol presented in[6]. Briefly, the series of composites were prepared in 96-well plate, sterilized with UV radiation for 20 min and washed four times with sterilized PBS. Materials tested were exposed to the bacteria (*S. aureus* or *E. coli*) cell suspension (50 µl per well) with a density of approximately 2.5 x $10^2$ and 6.5 x $10^2$ cells per well for *E. coli* and *S. aureus*. An empty well and the hydrogel from pristine chitosan crosslinked with 20mM genipin solution were used as the positive and the negative control, respectively. The plate was incubated for 4 h at 37 °C, next the liquid was gently mixed and the bacterial suspension was taken above the surface of the materials studied. The number of viable bacterial cells in the suspension was determined. For this purpose, the bacterial suspension was diluted in PBS, spread on MH agar medium and incubated at 37°C for approximately 24 hours. After that, the number of grown colonies were counted. Three independent experiments were performed.

**References**


1. Klara, J.; Marczak, A.; Łatkiewicz, A.; Horak, W.; Lewandowska-Łańcucka, J. Lysine-Functionalized Chondroitin Sulfate Improves the Biological Properties of Collagen/Chitosan-Based Injectable Hydrogels. *Int. J. Biol. Macromol.* **2022**, *202*, 318–331, doi:10.1016/j.ijbiomac.2022.01.026.

2. Kuljanin, J.; Jankovi, I.; Nedeljkovi, J.; Prstojevi, D.; Marinkovi, V. Spectrophotometric Determination of Alendronate in Pharmaceutical Formulations via Complex Formation with Fe(III) Ions. *J. Pharm. Biomed. Anal.* **2002**, *28*, 1215–1220, doi:10.1016/S0731-7085(02)00021-3.

3. Kokubo, T.; Takadama, H. How Useful Is SBF in Predicting in Vivo Bone Bioactivity? *Biomaterials* **2006**, *27*, 2907–2915, doi:10.1016/j.biomaterials.2006.01.017.

4. Gilarska, A.; Lewandowska-Łańcucka, J.; Horak, W.; Nowakowska, M. Collagen/Chitosan/Hyaluronic Acid – Based Injectable Hydrogels for Tissue

Engineering Applications – Design, Physicochemical and Biological Characterization. *Colloids Surfaces B Biointerfaces* **2018**, *170*, 152–162, doi:10.1016/j.colsurfb.2018.06.004.

5. Filipowska, J.; Lewandowska-Łańcucka, J.; Gilarska, A.; Niedźwiedzki, Ł.; Nowakowska, M. In Vitro Osteogenic Potential of Collagen/Chitosan-Based Hydrogels-Silica Particles Hybrids in Human Bone Marrow-Derived Mesenchymal Stromal Cell Cultures. *Int. J. Biol. Macromol.* **2018**, *113*, 692–700, doi:10.1016/j.ijbiomac.2018.02.161.

6. Gilarska, A.; Lewandowska-Łańcucka, J.; Guzdek-Zając, K.; Karewicz, A.; Horak, W.; Lach, R.; Wójcik, K.; Nowakowska, M. Bioactive yet Antimicrobial Structurally Stable Collagen/Chitosan/Lysine Functionalized Hyaluronic Acid – Based Injectable Hydrogels for Potential Bone Tissue Engineering Applications. *Int. J. Biol. Macromol.* **2020**, *155*, 938–950, doi:10.1016/j.ijbiomac.2019.11.052.
